# Advances in biomarkers for immunotherapy in small-cell lung cancer

**DOI:** 10.3389/fimmu.2024.1490590

**Published:** 2024-12-11

**Authors:** Hui Li, Peiyan Zhao, Lin Tian, Yuanhua Lu, Xinyue Wang, Wenjun Shao, Ying Cheng

**Affiliations:** ^1^ Medical Oncology Translational Research Lab, Jilin Cancer Hospital, Changchun, China; ^2^ Jilin Provincial Key Laboratory of Molecular Diagnostics for Lung Cancer, Jilin Cancer Hospital, Changchun, China; ^3^ Postdoctoral Research Workstation, Jilin Cancer Hospital, Changchun, China; ^4^ Department of Thoracic Oncology, Jilin Cancer Hospital, Changchun, China

**Keywords:** small-cell lung cancer, immunotherapy, biomarker, research advances, predictive, prognosis

## Abstract

Small-cell lung cancer (SCLC) is a refractory cancer with rapid growth and high aggressiveness. Extensive-stage SCLC is initially sensitive to chemotherapy; however, drug resistance and recurrence occur rapidly, resulting in a poor survival outcome due to lack of subsequently efficient therapy. The emergence of immune checkpoint inhibitors (ICIs) generated a new landscape of SCLC treatment and significantly prolonged the survival of patients. However, the unselected immunotherapy restrains both beneficiary population and responsive period in SCLC compared to the other tumors. The complex tumor origin, high heterogeneity, and immunosuppressive microenvironment may disturb the value of conventional biomarkers in SCLC including programmed cell death 1 ligand 1 and tumor mutation burden. Transcriptional regulator–based subtypes of SCLC are current research hotspot, revealing that Y (I) subtype can benefit from ICIs. Additionally, molecules related to immune microenvironment, immunogenicity, epigenetics, and SCLC itself also indicated the therapeutic benefits of ICIs, becoming potential predictive biomarkers. In this review, we discussed the advances of biomarkers for prediction and prognosis of immunotherapy, promising directions in the future, and provide reference and options for precision immunotherapy and survival improvement in patients with SCLC.

## 1 Introduction

Small-cell lung cancer (SCLC) accounts for 13%–15% of all lung cancers and is one of the deadliest refractory tumors with a 5-year survival rate of less than 7% (1, 2). On the basis of recent evidence, most common SCLC occurs in pulmonary neuroendocrine cells (PNECs), but, in some cases, it also occurs in lung epithelial cells, such as basal or club cells, and alveolar type 2 cells. Tuft cells, which are defined as chemosensory cells of the lung epithelial lining (3), also act as possible progenitor cells in a specific subtype of SCLC (4). Loss-of-function mutations including tumor protein 53 (TP53) and retinoblastoma 1 (RB1) in PNECs, tuft, club, or AT2 cells evidently evoke SCLC development. Moreover, SCLC may also transdifferentiate from lung adenocarcinoma following loss-of-driver mutations such as epidermal growth factor receptor (EGFR).

**Figure 1:**
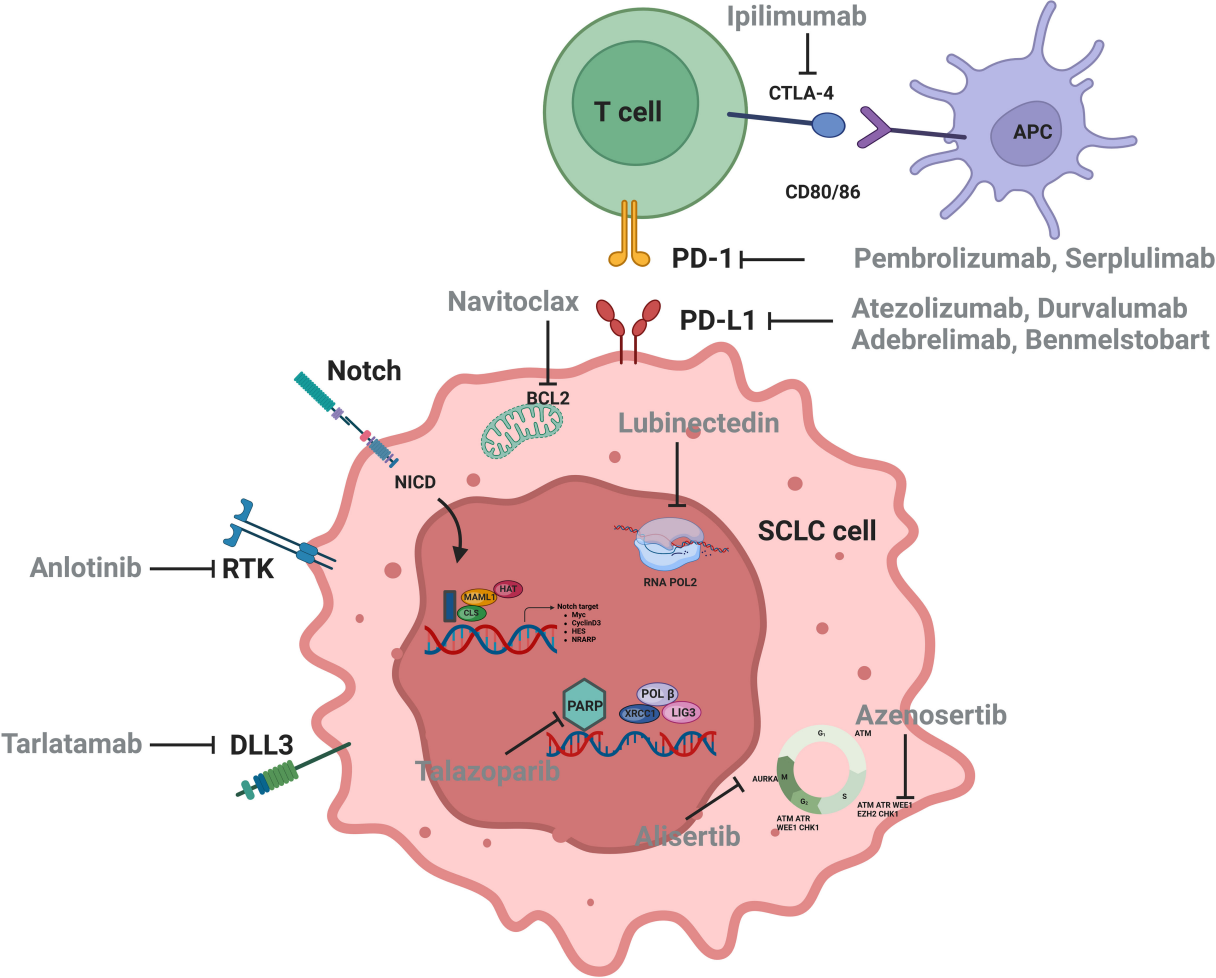
Signaling pathways and therapeutic targets relevant to SCLC.

SCLC is characterized by early metastatic spread, and two-thirds of patients showed tumor spreading beyond the chest at the time of initial diagnosis (2, 5). SCLC is sensitive to chemotherapy at first (6, 7) but relapses rapidly, making it an extremely challenging tumor type for oncologists (8). IMpower133 and CASPIAN set up a new milestone of immunochemotherapy in first-line extensive-stage SCLC (ES-SCLC) with prolonged progress-free survival (PFS) and overall survival (OS) (9–11). Food and Drug Administration (FDA) and National Medical Products Administration approved Atezolizumab and Durvalumab combination for first-line ES-SCLC treatment (9, 10, 12, 13). Subsequently, CAPSTONE-1 and ASTRUM-005 study refreshed the median OS (mOS) of ES-SCLC to more than 15 months by programmed cell death 1 ligand 1 (PD-L1) inhibitor Adebrelimab combined with chemotherapy (14) or programmed cell death protein 1 (PD-1) inhibitor Serplulimab combined with Etoposide and Carboplatin (EC) (15). Recently, combination of the PD-L1 inhibitor Benmelstobart, the angiogenesis inhibitor Anlotinib, and the chemotherapies significantly improved mOS up to 19.32 months (16). Innovative treatments for patients with SCLC are emerging (**Table 1**, **Figure 1**), but immunotherapy is still the first-line standard of care, which emphasizes the need of progress in immunotherapy for SCLC.

**Table 1:** Innovative therapeutic approaches for patients with SCLC.

Therapeutic approaches	Study name	Study phase	Mechanism of action	N	Outcomes
Benmelstobart, anlotinib, and etoposide/carboplatin	NCT04234607 Cheng et al. (17)	III	PD-L1 inhibitor, multitargeted anti-angiogenic agent, and chemotherapy	246	Benmelstobart and anlotinib plus EC vs. EC: mOS of 19.3 vs. 11.9 months (P = 0.0002), mPFS of 6.9 vs. 4.2 months (P < 0.0001), ORR of 81.3%, and mDoR of 5.8 months
Surufatinib plus toripalimab combined with etoposide and cisplatin	NCT04996771 Zhang et al. (18)	Ib/II	PD-L1 inhibitor, antiangiogenic therapy, and chemotherapy	35	ORR of 97.1%, DCR of 100%, mPFS of 6.9 months, and mOS of 21.1months
Durvalumab	NCT03703297 Cheng et al. (19)	III	PD-L1 inhibitor	264	mOS of 55.9 vs. 33.4 months and mPFS of 16.6 vs. 9.2 months
DS-7300 (I-DXd)	NCT05280470	I/II	B7-H3-targeted ADC	21	ORR of 52.4%, mDoR of 5.9 months, mPFS of 5.6, and mOS of 12.2 months
ABBV-706	NCT05599984	I	SEZ6-targeted ADC	23	ORR of 60.9%
PM8002 combined with paclitaxel	NCT05844150	II	PD-L1 and VEGF-A inhibitors	27	DCR of 81.8%, ORR of 72.7%, and mPFS of up to 5.5 months
Tarlatamab	NCT03319940 Paz-Ares et al. (20)	I	DLL3/CD3 BiTE	107	ORR of 23.4% and mDoR of 12.3 months
Lurbinectedin plus pembrolizumab	NCT04358237	I/II	Chemotherapy plus PD-1 inhibitor	28	ORR of 46.4%, mDoR of 11.4 months, mPFS of 5.3 months, and mOS of 11.1 months
Lurbinectedin plus atezolizumab	NCT04253145	I/II	Chemotherapy plus PD-L1 inhibitor	24	ORR of 57.69%, DCR of 84.61%, and mPFS of 4.93 months

PD-L1, programmed cell death-ligand 1; EC, etoposide and carboplatin; mOS, median overall survival; mPFS, median progression-free survival; ORR, objective response rate; mDOR, median duration of response; DCR, disease control rate; ADC, targeted antibody-drug conjugate; BiTE, bispecific T-cell bonding agent; PD-1, programmed cell death protein 1.

**Figure 2:**
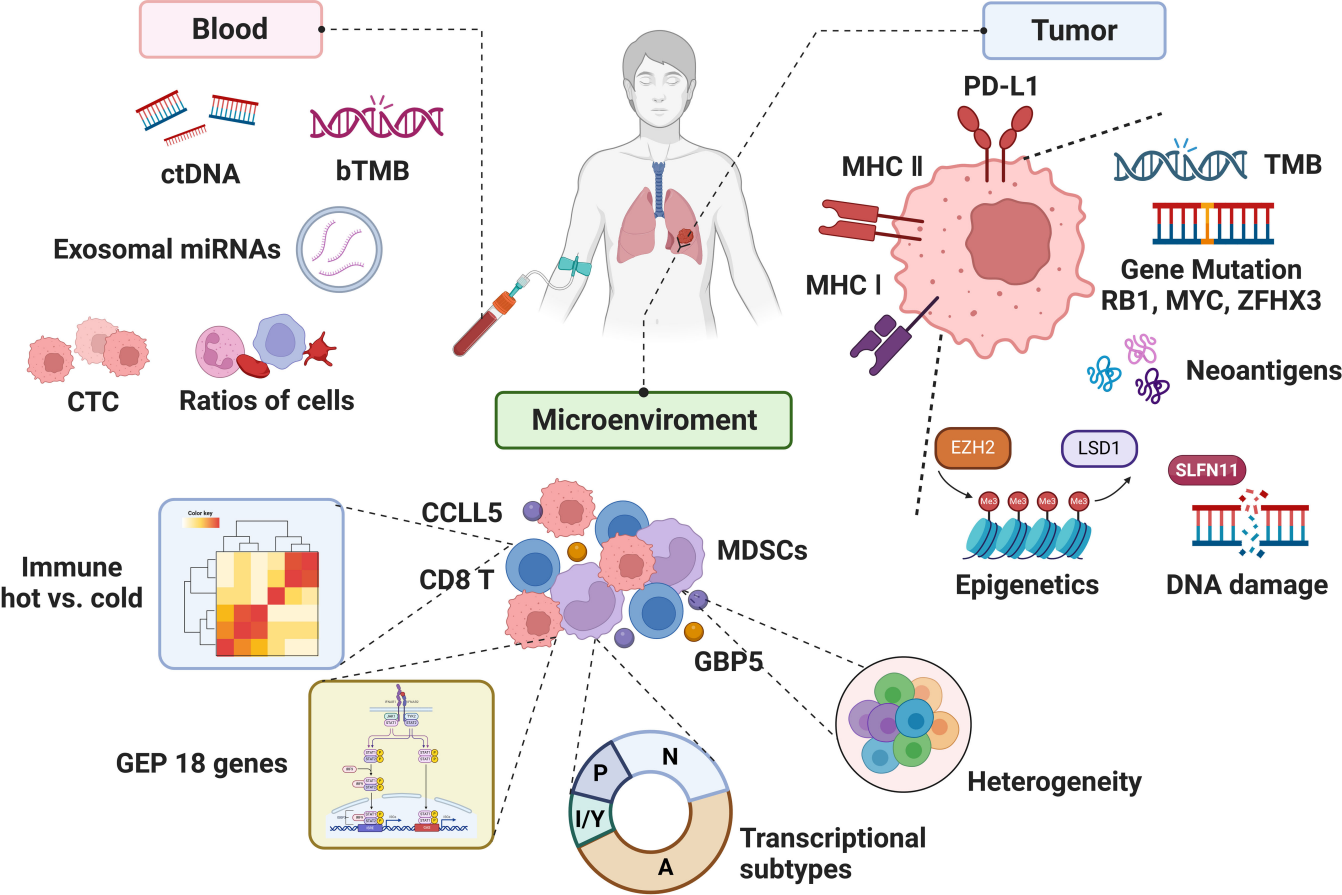
The biomarkers associated with the efficacy of immunotherapy in SCLC.

Although numerous studies have focused on biomarkers of immunotherapy in SCLC, the clinically applicable biomarkers in SCLC are still absence, partly due to the complexity of both tumor and microenvironment, lack of tumor specimens, etc. (2). With the understanding of the immune microenvironment and the rise of liquid biopsy, biomarker studies in SCLC immunotherapy may usher in new advances (**Figure 2**). Herein, we reviewed the progression, unmet clinical need, and future direction of biomarker studies to provide the conceptions for both SCLC clinical and translational research.

## 2 Tissue-derived biomarkers

### 2.1 Traditional biomarkers

#### 2.1.1 PD-L1

PD-L1 serves as a potential predictive marker for immune checkpoint inhibitors (ICIs) in patients with several solid cancers (21, 22), but it is lowly expressed in SCLC (23, 24). In CheckMate 032, the objective response rate (ORR) of the patients with tumor proportion score (TPS) of PD-L1 ≥1% was not significantly different from patients with TPS <1% (9.1% vs. 14.1% and 10% vs. 32.3%, respectively) in either Nivolumab group or Nivolumab + Ipilimumab group, revealing an irrelevance between PD-L1 expression and ORR (25). KEYNOTE-028 (n = 19) and KEYNOTE-158 (n = 64) showed that Pembrolizumab exhibited durable antitumor activity in a subset of patients with recurrent or metastatic SCLC, regardless of PD-L1 combined positive score (CPS) (26, 27), revealing that PD-L1 expression cannot predict the efficacy of ICIs in the ES-SCLC, whether assessed by TPS or CPS. Notably, a single-arm phase II study using Pembrolizumab as maintenance therapy after chemotherapy in patients with ES-SCLC showed that patients with positive stromal PD-L1 had higher partial response (PR) (37.5% vs. 8.3%), longer mPFS (6.5 vs. 1.3 months), and mOS (12.8 vs. 7.6 months) compared to those with negative PD-L1. Mechanistically, the expression of stromal PD-L1 might represent presence of restricted effector T cells in tumor microenvironment (TME), which was released by Pembrolizumab treatment, suggesting that stromal PD-L1 might be predictive for ICI response in patients with SCLC (28).

Similarly, in studies of the ICI + chemotherapy (ICI-combo) treatment in SCLC, PD-L1 has also been demonstrated to be an unpredictable biomarker (22, 29). IMpower133 reported that there was no significant difference in patients with positive PD-L1 expression (TPS ≥ 1% or CPS ≥ 1%) between the Atezolizumab + EC group and the EC group [9.7 vs.10.6 months; hazard ratio (HR), 0.87] with regard to mOS. Notably, in patients with negative expression of PD-L1, mOS was longer in the combination group than that in the EC group (10.2 vs. 8.3 months; HR, 0.51; P = 0.0150). Paradoxically, in patients with PD-L1 expression ≥5%, longer survival benefit was observed in the combination group than that in the EC group (21.6 vs. 9.2 months; HR, 0.60; P = 0.2527) (30). However, CASPIAN showed that negative PD-L1 impacted the OS neither in the Durvalumab combined with Etoposide plus Cisplatin (EP) nor EP group (P = 0.54 and P = 0.23, respectively) (31). Similarly, ASTRUM-005 found that PD-L1 expression was not related to mOS both the Serplulimab group and the placebo group (P = 0.44) (15). The reason why TPS or CPS could not reliably predict the efficacy of ICI-combo for SCLC treatment was partly attributed to the limitations such as substantial heterogeneity among patients with SCLC (**Table 2**). A more comprehensive understanding of the TME is warranted to develop a composite scoring system to evaluate PD-L1 expression across multiple components of the TME in SCLC.

**Table 2:** Studies of traditional biomarkers of SCLC treated with immunotherapy.

Clinical trial	Phase	N	Setting	Experimental arm	Primary endpoint	Biomarker	Cutoff	Detection method	Positive rate of PD-L1	ORR	Median PFS	Median OS	Reference
CheckMate 032	Phase 1/2	216	Third or later line	Nivolumab; Nivolumab + Ipilimumab	ORR	PD-L1	TPS ≥ 1%	Dako 28-8 mAb	18% (21/116)	PD-L1 ≥ 1% vs. TPS < 1% Nivo: 9.1% vs. 14.1%; Nivo + Ipi: 10% vs. 32.3%	NA	NA	(25)
						TMB	Low: <143 mutations; medium: 143–247 mutations; high: ≥248 mutations	Tissue, WES	NA	NA	1.3 vs. 1.3 vs. 1.4 months	3.1 vs. 3.9 vs. 5.4 months	(25)
KEYNOTE-028	Phase 1	19	Third or later line	Pembrolizumab	ORR	PD-L1	TPS ≥ 1%	Dako 22C3mAb	31.7% (46/145)	PD-L1 positive: 33.3%	PD-L1 positive: 1.9 months	PD-L1 positive: 9.7 months	(26, 27)
KEYNOTE-158	Phase 2	64	Third or later line	Pembrolizumab	ORR	PD-L1	CPS ≥ 1%	Dako 22C3mAb	39% (42/107)	18.7% vs. 35.7%	1.9 vs. 2.1 months	5.9 vs. 14.9 months	(26)
	Phase 2	45	Maintenance treatment after chemotherapy	Pembrolizumab	PFS	PD-L1	Positive stromal PD-L1 vs. negative PD-L1	Dako 22C3mAb	10% (3/30) (TC), 40% (8/20) (stromal tissue)	PR rate: 37.5% vs. 8.3%	6.5 vs. 1.3 months	12.8 vs. 7.6 months	(28)
IMpower133	Phase 3	403	First-line	Atezolizumab + EC; Placebo + EC	PFS and OS	PD-L1	TPS ≥ 1% or CPS ≥ 1% PD-L1 ≥ 5%	SP263	TC: 5.8% (8/137) IC: 50.4% (69/137)	NA	NA	PD-L1 ≥ 1%: 9.7 vs.10.6 months PD-L1<1%: 10.2 vs. 8.3 months PD-L1 ≥ 5%: 21.6 vs. 9.2 months	(30)
						TMB	10 mut/Mb and 16 mut/Mb	Blood, FoundationACT	NA	NA	NA	bTMB<10: 11.8 vs. 9.4 months; bTMB ≥ 10: 14.9 vs. 11.2 months; bTMB<16: 12.5 vs. 10.o months; bTMB ≥ 16: 17.1 vs. 11.9 months	(30)
CASPIAN	Phase 3	805	First-line	Durvalumab + EP; EP	OS	PD-L1	TPS or CPS ≥ 1%	SP263	TPS: 5.1% (14/227) CPS: 23.4% (62/22737)	NA	NA	PD-L1 positive: HR, 0.46; 95% CI, 0.119–1.793 PD-L1 negative: HR, 0.66; 95% CI, 0.491–0.896	(31)
						TMB	NA	Tissue, FoundationOne CDx	NA	TMB was not predictive of Durvalumab ± Tremelimumab + EP vs. EP			(32)
KEYNOTE-604	Phase 3	453	First-line	Pembrolizumab + EP; Placebo + EP	PFS and OS	PD-L1	CPS ≥ 1%	Dako 22C3mAb	40.8% (185/453)	NA	Positive HR, 0.67; 95% CI, 0.49–0.92; negative HR, 0.72; 95% CI, 0.53–0.96	PD-L1 positive: HR, 0.84; 95% CI, 0.60–1.18; PD-L1 negative: HR, 0.80; 95% CI, 0.58–1.15	(33)
ASTRUM-005	Phase 3	585	First-line	Serplulimab + EC; Placebo + EC	OS	PD-L1	TPS ≥ 1%	Dako 22C3mAb	Serplulimab: 62/389 Placebo: 34/196	NA	NA	PD-L1 positive: NR vs. 12.9 months; HR, 0.92; 95% CI, 0.44–1.89. PD-L1<negative: 15.0 vs. 10.5 months; HR, 0.58; 95% CI, 0.44–0.76.	(15)

PD-L1, programmed cell death-ligand 1; TMB, tumor mutation burden; bTMB, blood TMB; ORR, objective response rate; PFS, progression-free survival; OS, overall survival; WES, whole-exome sequencing; TPS, tumor proportion score; CPS, Combined Positive Score; TC, tumor cell; IC, immune cell; EP, Etoposide plus Cisplatin; EC, Etoposide plus Carboplatin; HR, hazard ratio. NA, not applicable.

#### 2.1.2 TMB

The genetic landscape of tumor serves as the primary factors affecting tumor response to ICIs (34). Several studies identified the frequently altered genes in SCLC (35, 36) (**Table 3**), and gene losses and mutations, including the deletion of TP53 and RB1 and amplification of SOX2 and MYC in SCLC, lead to the high tumor mutation burden (TMB) in SCLC (37). A study of American Society of Clinical Oncology (ASCO) 2017 revealed that the median SCLC TMB was 9 mut/Mb, and the 90th percentile TMB was 19.6 mut/Mb (38). High TMB of SCLC contributes to a large number of potential tumor-specific antigens, which may imply the effectiveness of immunotherapy in SCLC (39).

**Table 3:** Frequently altered genes in SCLC.

Gene	Main function	Alteration	Frequency in SCLC (%)
TP53	Tumor suppressor; stress response; transcription regulation	Inactivation mutation; deletion	95
RB1	Tumor suppressor; cell cycle regulation; transcription repression	Inactivation mutation; deletion	80
MYC paralogs	Cell proliferation; metabolism	Amplification; overexpression	6–25
KMT2D	Tumor suppressor; histone modification; chromatin remodeling	Inactivation mutation; deletion	13
PTEN	Tumor suppressor; PTEN-mTOR signaling pathway	Inactivating mutation; deletion	7
PIK3CA	Oncogene; PTEN-mTOR signaling pathway; PI3K signaling pathway	Activating mutation	7
NOTCH1	Tumor suppressor; cell-cell signaling	Inactivating mutation	6
RICTOR	PTEN-mTOR signaling pathway	Amplification	5.6
CREBBP	Tumor suppressor; acetyltransferase; chromatin remodeling; transcription regulation	Inactivating mutation; deletion	5
APC	Tumor suppressor; WNT signaling pathway	Inactivating mutation; deletion	4
EGFR	Oncogene; RAS signaling pathway	Activating mutation	4
FAT1	Tumor suppressor; cell-cell signaling	Inactivating mutation; deletion	4
NF1	Tumor suppressor; RAS signaling pathway	Inactivating mutation; deletion	4
ARID1A	Tumor suppressor; chromatin remodeling; transcription regulation	Inactivating mutation; deletion	3
KEAP1	Tumor suppressor; tumorigenesis	Inactivating mutation; deletion	3
KRAS	Oncogene; RAS signaling pathway	Activating mutation	3
NOTCH3	Tumor suppressor; cell-cell signaling	Inactivating mutation; deletion	3
PTPRD	Tumor suppressor; chromatin remodeling	Inactivating mutation; deletion	3
EP300	Tumor suppressor; chromatin remodeling	Inactivating mutation; deletion	2
STK11	Tumor suppressor; immunotherapy; genetic correlation	Inactivating mutation; deletion	1.7

The predictive significance of TMB for immunotherapy varied widely across different clinical trials (**Table 2**). CheckMate 032 firstly investigated the predictive value of TMB for SCLC immunotherapy. Patients with SCLC (n = 211) receiving Nivolumab or Nivolumab + Ipilimumab were divided into three groups according to the TMB cutoff values of 143 mut/Mb and 247 mut/Mb. The mOS and mPFS in low-, medium-, and high-TMB groups were 3.1 vs. 3.9 vs. 5.4 months and 1.3 vs. 1.3 vs. 1.4 months, respectively (25), suggesting the predictive effect of high TMB for immunotherapy benefit. KEYNOTE-028 (40) retrospectively discovered that higher TMB was significantly correlated with ORR and longer PFS (P = 0.018 and P = 0.051) in patients (n = 77) treated with Pembrolizumab. Similarly, KEYNOTE-158 (41) retrospectively found that patients with high TMB (≥10 mut/Mb) treated with ICIs had higher ORR (28.3%; 95% CI, 20.5–37.3) and improved survival time, also suggesting that TMB may serve as a potential biomarker for ICI therapy in SCLC.

Controversially, CASPIAN study retrospectively approved no correlation between TMB and clinical efficacy (OS, PFS, or ORR) (32) detected by FoundationOne CDx assay from 805 tumor tissue derived from patients with ES-SCLC treated with Etoposide and Cisplatin (EP), Durvalumab + EP, or Durvalumab + Tremelimumab + EP, presenting a challenge for TMB as a predictive biomarker of ICI-combo in SCLC.

Considering the difficulty of acquiring tumor tissue from patients with ES-SCLC and high tumor heterogeneity, researchers proposed to assess blood TMB (bTMB). In IMpower133, bTMB was evaluated in 346 patients, and the cutoff was determined as 10 mut/Mb and 16 mut/Mb, respectively. There was no significant difference in mOS between the ICI-combo and chemotherapy groups regardless of different cutoff (30), possibly due to changes in the nucleotide pool of tumor cells caused by chemotherapy, which would weaken the predictive effect of TMB on the outcome of ICIs (42). The application of bTMB was limited by timeliness of sample testing and sensitivity of testing technology. Based on the above, neither tissue TMB nor bTMB could accurately predict the efficacy of ICI-combo in SCLC. Complex detection technology, high cost, and unclear definition of thresholds limit the utilization of TMB as a reliable predictive biomarker; thus, prospective clinical studies are needed to verify its feasibility for predicting the efficacy of ICI-combo in SCLC in the future.

### 2.2 Biomarkers in exploration

Because traditional immunotherapy biomarkers, such as PD-L1 and TMB, cannot predict the efficacy of SCLC immunotherapy, researchers are exploring more new biomarkers that can be used. Some studies have demonstrated that alterations in a single gene or protein in SCLC may be associated with the benefit of immunotherapy in SCLC (**Table 4**). However, with the rise of omics or even multi-omics studies, it seems more reasonable to use scores composed of multiple gene or protein signatures to predict efficacy, representing more characteristics of SCLC, which has become the main direction of the biomarker studies in SCLC immunotherapy (**Table 5**).

**Table 4:** Tissue-derived biomarkers in exploration for immunotherapy in SCLC.

Biomarker	Detection method	Treatment	N	Cutoff	ORR	Median OS	Median PFS	Reference
RB1	WES	ICI or ICI-combo	42	NA	NA	RB1-mu vs. RB1-wt: 5 vs. 23.1 months, P = 0.04	NA	(43)
RB1	WES	Nivolumab	460	NA	NA	RB1-wt patients had longer OS (HR, 1.41; P = 0.041)	NA	(43)
MYC	RNA targeted sequencing	Chemo alone or ICI-combo	135	Median value for MYC expression	NA	NA	MYC-low vs. MYC high ICI-combo: 5.3 vs. 4.0 months, P = 0.028; HR, 2.18 Chemo: P = 0.77; HR, 1.09	(44)
SLFN11	IHC	Atezoluma± Talazoparib as maintenance therapy	106	H-score ≥ 1	NA	NA	4.2 vs. 2.8 months, P = 0.056	(45)
MHC-I	IHC	ICI	31	Median H-score + 2.5 interquartile ranges	NA	MHC-I-high had longer OS, P < 0.01)	NA	(46)
MHC-II	FoundationOne CDx	Durvalumab + Tremelimumab + EP	142	NA	NA	14.9 months (95% CI, 10.4–21.2) vs. 10.5 months (95% CI, 7.6–12.9); HR, 0.59	NA	(47)
APM-related genes	RNA sequencing	Nivolumab	286	NA	NA	High APM expression was significantly associated with the OS benefit (P = 0.000032)	NA	(48)
Neoantigen load	WES	ICI-combo	135	Median	NA	High neoantigen load had higher 12-month PFS rate, 16.1% vs. 0%	NA	(44)
LSD1	RNA sequencing	Nivolumab combination or Nivolumab alone	286	NA	NA	High expression of LSD1 was significantly associated with worse OS (P = 0.02)	NA	(48)
CD8+ TIL	IHC		286	≥1%	NA	Patients with CD8-positive (≥1%) tumors showed improved OS (HR, 0.51) in the Nivolumab group Nivolumab + Ipilimumab group (HR, 0.7; 95% CI)	NA	(48)
MDSCs	RNA- sequencing	Pembrolizumab/placebo + chemotherapy	158	<Median value corrected for GEP genes	NA	Monocyte MDSCs: 15.5 vs. 8.3 months Granulocyte MDSCs: 16.3 vs. 8.3 months	NA	(33)
GBP5	IHC	ICI	35	NA	GBP5-high had higher proportion of responders (P < 0.05)	NA	NA	(49)
CCL5	RNA sequencing data	Immunotherapy	159	4.77	NA	High CCL5 expression indicated longer OS in patients with SCLC (P < 0.0001) accepting immunotherapy (P = 0.032)	NA	(50)

WES, whole-exome sequencing; ORR, objective response rate; PFS, progression-free survival; OS, overall survival; IHC, immunohistochemistry; ICI, immune checkpoint inhibitor; ICI-combo, ICI + chemotherapy; chemo, chemotherapy; HR, hazard ratio; tumor-infiltrating lymphocyte (TIL); GEP, gene expression profile; APM, antigen processing and presenting machinery; MDSC, myeloid-derived suppressor cells. NA, not applicable.

**Table 5:** Biomarkers based on multi-omics profiling for SCLC immunotherapy.

Biomarker	Detection method	Treatment	N	Cutoff	ORR	Median OS	Median PFS	Reference
SCLC-I	RNA sequencing	Atezolizumab/placebo + EP	276	NA	NA	Achieved the longest median OS after Atezolizumab + EP treatment (18.2 vs. 10.9 vs. 10.6 vs. 9.6 months)	NA	(51)
T-eff–high/TAM-low NE tumors	RNA sequencing	Atezolizumab plus EC	271	Median cohort-wide expression	NA	HR, 0.26 (95% CI, 0.12–0.57)	NA	(52)
SCLC-Y/I	RNA sequencing	Durvalumab + EP; EP	104	Higher expression level than other Transcription factor	NA	10.4 vs. 0.8 vs. 2.4 months vs. no benefit 6.3 vs. 1.2 vs. 4.1 months vs. no benefit	NA	(53)
POU2F3 expression	IHC	ICI treatment	28	H-score > 90	Increased ORR (AUC = 0.813)	Prolonged OS (P = 0.022)	NA	(54)
ZFHX3 mutation	WES	PD-1/PD-L1 blockade combined with chemotherapy	12	NA	All three patients with ZFHX3 mutation (100%) belonged to MPR, whereas only two patients (22%) had MPR in wild-type patients (P = 0.045)	NA	NA	(55)
GEP expression	RNA sequencing	Pembrolizumab combined chemotherapy	159	>1st tertile values	NA	Combination: P = 0.003 Chemo: P = 0.0002	Combination: P = 0.002 Chemo: P = 0.001	(33)
Immune hot/cold	Targeted transcriptomic sequencing	Anti–PD-1 treatment	14	Expression of 53 DEGs	Patients with “immune hot” features tended to benefit more from ICI than the other patients with “immune cold” SCLC	NA	NA	(56)

ORR, objective response rate; PFS, progression-free survival; OS, overall survival; HR, hazard ratio; EP, Etoposide plus Cisplatin; EC, Etoposide plus Carboplatin; ICI, immune checkpoint inhibitor; IHC, immunohistochemistry; T-eff, effector T cell; TAM, tumor-associated macrophage; NE, neuroendocrine; WES, whole-exome sequencing; PD-1, programmed cell death protein 1; PD-L1, programmed cell death-ligand 1; MPR, major pathologic response; DEG, differentially expressed genes. NA, not applicable.

#### 2.2.1 Single biomarker

##### 2.2.1.1 RB1

RB1 inactivation is obligatory in SCLC (5) and loss of RB1 not only can cause tissue cancerous but also can lead to impaired immune response and significant attenuation of genes related to immune function, suggesting the relationship between RB1 and immune characteristics (57), whereas wild-type (WT) RB1 was a surrogate marker for yes-associated protein 1 (YAP1) expression in SCLC and related to the survival and chemotherapy responsiveness of patients (58). Moreover, the effect of RB1 alterations on the immune microenvironment in SCLC was verified when adding high expression of YAP1 and enriched interferon gamma (IFN-γ), T-cell response, and human leukocyte antigen (HLA)-related genes (59). In a retrospective cohort of 42 patients receiving ICIs or ICI combination therapy, patients with RB1 WT had significantly longer mOS (5 vs. 23.1 months, P = 0.04) and enriched immune-related genes and immune rejection phenotypes, compared to patients with RB1 mutation. In CheckMate 032, patients (n = 460) with RB1 WT after Nivolumab treatment had significantly improved prognosis, compared with those with RB1 mutation (HR, 1.41; P = 0.041) and longer OS in patients with low RB1 loss of function feature scores (43), suggesting RB1 mutation status as a potential biomarker for SCLC immunotherapy.

##### 2.2.1.2 MYC

MYC is involved in the recruitment of elements in TME, including making the stroma more suitable for tumor cell progression, facilitating immune evasion and promoting tumor to a more aggressive and metastatic phenotype (60–63). In SCLC, MYC activated Notch to dedifferentiate neuroendocrine (NE) tumor cells and promoted the temporal evolution of SCLC from ASCL1^+^ state to NEUROD1^+^ state to YAP1^+^ state (64). Considering the immune infiltration profile and the potential benefit of YAP1^+^ subtype, MYC may be a predictive biomarker for SCLC immunotherapy.

Kazuhiko et al. (44) retrospectively studied the efficacy of chemotherapy alone or ICI-combo in 135 patients with ES-SCLC, who were stratified into “inflamed tumor” and “non-inflamed tumor” groups based on both PD-L1 (≥1%), positive score (CPS), and CD8^+^ tumor-infiltrating lymphocyte (TIL) density (≥85/mm^2)^ and found that MYC (P = 0.02) and SOX11 (P < 0.001) were the two most upregulated genes in “non-inflamed tumors” compared to those in “inflamed tumors,” suggesting that MYC has weak immunoreactivity in SCLC. Furthermore, in the ICI-combo cohort (n = 39), the patients with low MYC expression had longer mPFS (5.3 vs. 4.0 months; P = 0.028; HR, 2.18) and higher 12-month PFS rates (23.5% vs. 4.6%) than that with high MYC expression, whereas no difference was found in chemotherapy cohort (n = 50; P = 0.77; HR, 1.09), indicating MYC expression as a predictive biomarker. Mechanistically, the study revealed that oncogenic activation of MYC impaired IFN-γ–mediated transcriptional activation by downregulating JAK2 (65), suggesting the potential of MYC as a predictive biomarker of SCLC immunotherapy, which needed to be supported by more studies on the mechanism of MYC regulating SCLC immunity.

##### 2.2.1.3 SLFN11

SLFN11 is a DNA/RNA helicase and can be recruited to DNA damage sites and regulates replication stress (66). Pietanza et al. reported that SLFN11 is predictive for both improved PFS and OS for Veliparib in SCLC; patients with SLFN11-positive tumors treated with TMZ/Veliparib had significantly prolonged PFS (5.7 vs. 3.6 months, P = 0.009) and OS (12.2 vs. 7.5 months, P = 0.014) (67, 68); thus, it is considered as a biomarker for poly ADP-ribose polymerase (PARP) inhibitor (PARPi). In 2023, SWOG S1929 reported at ASCO that 79% of SCLC tumor tissues were SLFN11-positive, and, in patients with SFN11-positive (H-score ≥ 1) ES-SCLC, addition of PARPi Talazoparib to Atezolumab as maintenance therapy following first-line chemotherapy + Atezolumab improved mPFS (4.2 vs. 2.8 months, P = 0.056) (45), demonstrating that SLFN11 might be a predictive biomarker for the efficacy of ICIs combined with PARPi treatment.

##### 2.2.1.4 Major histocompatibility complex molecules

Major histocompatibility complex (MHC) molecules present antigen fragments to the immune system and also are expressed on tumor cells (69). MHC class I (MHC-I) is an important component of adaptive immune system, indiscriminately presenting tumor antigens to cytotoxic T lymphocytes recognition (69, 70). However, in SCLC, low expression of MHC-I (71, 72) was correlated with less immune infiltration (56), suggesting the relationship between MHC-I and immune characteristics. Moreover, multiplexed immunofluorescence detection of SCLC samples revealed that more CD3^+^ T cells and CD45^+^/PD-L1^+^ immune cells in the intra-tumoral region in high–MHC-I expression SCLC tissue and low-NE differentiation, compared to that with low MHC-I expression. Patients with high MHC-I expression (n = 7) had long-term response to ICI treatment and longer OS than patients with low expression level (n = 24, P < 0.01) (46).

MHC-II molecules are mainly presented on CD4^+^ T cell (73). CASPIAN study found that the incidence of MHC-II allele HLA-DQB1*03:01 was 37%, and the mOS of allele-positive patients was significantly longer than that of negative patients in Durvalumab (D) + Tremelimumab (T) + EP treatment cohort [14.9 months (95% CI, 10.4–21.2) vs. 10.5 months (95% CI, 7.6–12.9); HR, 0.59], which was not found in D + EP (HR, 0.93) and EP cohorts (HR, 0.94) (47). Consistently, highly heterozygous MHC sites were correlated with significantly improved OS after ICI treatment (P = 0.003; HR, 2.03) (74), demonstrating the potential of MHC expression as a predictive biomarker for ICI treatment in SCLC.

Antigen presentation was known to be inhibited in most patients with SCLC, which may account for the low ICI response rate of SCLC with high TMB (71, 75). Checkmate-032 demonstrated that the expression of antigen-processing and -presenting machinery (APM)–related gene, including HLA-A, HLA-B, HLA-C, B2M, TAP1, and TAP2, was evaluated to predict benefit of ICI. High APM expression was significantly associated with the OS benefit of Nivolumab treatment (P = 0.000032) but not associated with that of Nivolumab + Ipilimumab treatment (48, 76).

##### 2.2.1.5 Neoantigens

Neoantigens are novel antigens generated by tumor cells due to various tumor-specific alterations, including genomic mutations, dysregulated RNA splicing, disrupted post-translational modifications, and integrated viral open reading frames (77). As non-self antigens, neoantigens are likely to trigger responses of T and B cells and ultimately identified by calculating the frameshift mutation, splicing variants, gene fusion, gene expression, and other variants of MHC through next-generation sequencing (NGS) combined with proteomic sequencing. Above all, frameshift mutations by insertion or deletion (fsindels) were considered to generate more immunogenic tumor-specific neoantigens, resulting in a better response to ICIs (78), in several cancers (79). Recently, Shen et al. (80) analyzed the pre-treatment blood samples by microarrays of frameshift peptides assay from 57 patients with NSCLC and nine patients with SCLC receiving ICIs and ICI-combo and found high predictive accuracy of 97.8% for disease progression (80). In a retrospective study based on the whole-exome sequencing (WES), Kanemura et al. found that, among 135 patients with ES-SCLC, patients with high neoantigen load (n = 26) had higher 12-month PFS rate than those with low neoantigen load (n = 18) (16.1% vs. 0%) in the ICI-combo cohort, and there was a correlation between high frameshift neoantigen load with antigen presentation and co-stimulatory signaling (44). Therefore, neoantigens with high immunogenicity and tumor specificity may serve as predictive biomarkers of ICI response in SCLC.

##### 2.2.1.6 Epigenetic characteristic

SCLC tissues or cell lines exhibited a diversity of epigenetic abnormalities (81). Histone demethylase LSD1, a key determinant of MHC-I expression and antigen presentation in SCLC, could remove monomethylation and dimethylation of H3K4 and H3K9 to promote gene expression (82, 83). Enhancer of zeste homolog (EZH2), an enzymatic subunit of Polycomb Repressive Complex 2, could catalyze trimethylation of H3K27 to silence genes, whereas the inhibition of EZH2 induced MHC-I expression and immune responses in preclinical models of advanced NE tumors, including SCLC (46, 84, 85). In CheckMate 032, for both Nivolumab and combination cohort, a high expression of LSD1 was significantly associated with worse OS (P = 0.02), and same trend was found for EZH2 (P = 0.27) (48), opening up the understanding of epigenetic modification predicting the efficacy of immunotherapy in SCLC.

##### 2.2.1.7 Immune cell infiltration

SCLC was characterized as an immune cold tumor, with infiltrating immune cells comprising only one-fifth of those in NSCLC (86), particularly cytotoxic T cells, leading to lower response rate to ICI therapy in SCLC compared to that in NSCLC (87).

Survival of patients with SCLC is correlated with higher expression levels of classical surface biomarkers including CD3, CD20, and CD45 on TIL (88) but negatively related with Foxp3+ cells (89). CTLA-4 and PD-1/PD-L1–based immunotherapy primarily exerted anti-tumor effects by modulating the activation and proliferation of T cells (90), especially regulatory T cells (Tregs). The lack of reporting of the proportion of infiltrating Tregs in SCLC tumor tissues led to the inability to determine whether Tregs disrupted the efficacy of ICI therapy in SCLC. However, the proportion of Tregs in the peripheral blood of patients with SCLC was lower than that of patients with NSCLC (9.0 ± 1.2 vs. 15.0 ± 3.9), and the combination of chemoradiotherapy and biological therapy significantly reduced the proportion of Tregs in peripheral blood (9.0 ± 1.2 vs. 7.6 ± 1.1) (91), confirming the relationship between Tregs and immunotherapy in SCLC, which is worthy of further exploration. Moreover, a subtype of SCLC with non-NE features had heightened inflammatory gene signatures and immune cell infiltration, potentially suggesting better response to ICIs (56).

CheckMate 032 revealed that patients with SCLC with CD8-positive (≥1%) tumors showed improved OS (HR, 0.51) in the Nivolumab group, and a similar trend was observed in the Nivolumab + Ipilimumab group (HR, 0.7; 95% CI) (48). Myeloid-derived suppressor cells (MDSCs) are generally recognized as suppressor cells involved in self-tolerance and immune homeostasis, leading to an immunosuppressive microenvironment and tumor progression. KEYNOTE-604 explored that patients with low infiltration level of monocyte and granulocyte MDSCs who received Pembrolizumab + chemotherapy exhibited longer OS than patients with high infiltration levels (15.5 vs. 8.3 months and 16.3 vs. 8.3 months, respectively) (33). This discovery presents a novel perspective, and future studies might focus on exploring the infiltration patterns of diverse immune cells and non-immune cell components of SCLC.

##### 2.2.1.8 GBP5

Guanylate-binding protein-5 (GBP5), the IFN-inducible guanine nucleotide triphosphate hydrolysis (GTPases) (92), played a key role in innate immune system inflammation and macrophage activation (93). Patients with GBP5-high SCLC had highly expressed cytotoxicity, chemokines, antigen-presenting, and TNF family–related genes and higher proportion of responders to immunotherapy than those in the GBP5-low group (P < 0.05), indicating the potential of CBP5 to predict ICI efficacy for patients with SCLC (49).

##### 2.2.1.9 CCL5

CC chemokine ligand 5 (CCL5) and CC chemokine receptor 5 (CCR5) were not only the leading players in tumor progression (94, 95) but also exerted anti-tumor immunity by recruiting T cells and dendritic cells, enhancing immunotherapy responses of multiple tumors (94). In SCLC mouse models, increased CCL5 enhanced response to ICI (96). Moreover, high CCL5 expression indicated longer OS in patients with SCLC (P < 0.0001), accepting immunotherapy (P = 0.032) (50). Up to now, studies on inflammatory factors as biomarkers of immunotherapy efficacy or stratified therapy are still limited and require further investigation in SCLC.

#### 2.2.2 Biomarkers based on multi-omics profiling

##### 2.2.2.1 Transcriptional subtypes

In SCLC, the dysregulation of prevalent transcriptional factors, as revealed in genomics studies, underscores the pivotal role of transcriptional regulation (5, 97, 98). Rudin et al. initially classified SCLC into four transcriptional subtypes based on the expression of key transcription factors, achaete-scute homolog 1 (ASCL1), neurogenic differentiation factor 1 (NEUROD1), POU class 2 homeobox 3 (POU2F3), and YAP1, using RNA-seq data (98). Then, Gay et al. utilized non-negative matrix factorization to cluster RNA-seq data from SCLC tissues and identified a distinct SCLC-I subtype with lower expression of ASCL1, NEUROD1, and POU2F3, which possessed inflammatory/interstitial properties (51). Both SCLC-Y and -I subtypes exhibit a non-NE phenotype, which has been defined as “immune oasis” for the greater immune cell infiltration and checkpoint molecule expression (99). Owonikoko et al. (59) distinguished the inflammatory characteristics of SCLC-Y subtype, including high expression of IFN-γ response genes, HLA genes, T-cell receptor genes, and increased T-cell inflamed gene expression profile (GEP) score compared to other subtypes. Patients with SCLC-Y subtype displayed trends toward prolonged mOS compared to patients with other three subtypes (20.1 vs. 14 vs. 16.7 vs. 8.1 months). Moreover, the higher rate of YAP1-positive expression detected by immunohistochemistry (IHC) in ES-SCLC than that in LS-SCLC (30.6% vs. 8.5%, P = 0.0058) in the validation cohort might suggest a correlation between YAP1 expression and improved prognosis.

On the basis of the discovery of transcriptional subtypes of SCLC, the therapeutic vulnerability of different subtypes has also been intensively studied. SCLC-A subtype was found to highly express MYCL and DLL3 (100). Although the DLL3-targeting antibody-drug conjugate (ADC) drug rovalpituzumab tesirine (Rova-T) has not been shown to be superior to topotecan in OS in patients with relapsed and/or recurrent SCLC (101), the DLL3-targeting bispecific T-cell engager Tarlatamab effectively promoted regression of SCLC tumors and liver metastases in mouse models (102). In addition, a phase I trial showed that Tarlatamab had an ORR of 23.4% and a median duration of response (mDOR) of 12.3 months in patients with relapsed and/or recurrent SCLC who had received a median of two prior therapies, making it a promising option with an acceptable safety profile (20). In DeLLphi-301 study, ORR was 40% or 32%, and mPFS was 4.9 or 3.9 months for patients with SCLC who had received a median of two prior therapies in the 10-mg or 100-mg Tarlatamab groups, respectively (103). On the basis of these promising results, FDA granted accelerated approval of Tarlatamab (10 mg) for patients with ES-SCLC with disease progression during or after platinum-based chemotherapy in May 2024. Other DLL3-targeting bispecific T-cell engagers, such as BI-764532 and HPN328, that enhanced the activity of CD4^+^ and CD8^+^ T cells against DLL3-expressing SCLC in mice (104, 105), are being studied. BCL-2 is a direct transcriptional target of ASCL1 and overexpressed in SCLC-A. In preclinical models of SCLC, combined inhibition of BCL-2 or BCL-XL and MCL1 showed synergistic activity (106, 107), but BCL-2 inhibition failed to improve the clinical efficacy of standard chemotherapy (108, 109).

Studies showed that SCLC-N subtype is associated with MYC overexpression, and patients with SCLC-N may benefit from therapies such as CHK1 inhibitors (110), pegylated arginine deiminase (111) and Aurora A kinase inhibitors (112). A subset of patients with MYC expression showed significantly improved PFS with Aurora A kinase inhibitor, alisertib plus paclitaxel, revealing that MYC expression may be potential predictive biomarkers of alisertib efficacy (112). Currently, a trial of MRT-2359 in patients with SCLC harboring MYCN or MYCL alterations (NCT05546268) is ongoing (113). MRT-2359 is an effective oral G1 to S phase transition 1 (GSPT1) degrader that indirectly targets MYC with preferential antiproliferative activity against MYC-driven lung cancer.

SCLC cells overexpressing POU2F3 rely on the lineage transcription factors SOX9, ASCL2, and insulin-like growth factor 1 receptor (IGF-1R), suggesting potential vulnerability to tyrosine kinase inhibitors (4). Patients with SCLC-P subtype tumors showed the worst OS and drug response data in subgroup analysis of IMpower133, implying that SCLC-P cells were sensitive to PARPis (51). While neither PARPis (67, 114) nor IGF-1R inhibitors (115, 116) have shown clinical benefit in patients with ES-SCLC, it may be worthwhile to test these drugs in patients with SCLC-P.

YAP1 is a key molecule in Hippo signaling pathway, and SCLC-Y cells were found to be resistant to Irinotecan and BCL-2 inhibitors. A preclinical study showed that SCLC-Y subtype cells were sensitive to mTOR and PLK inhibitors (117). Several preclinical and clinical studies identified that SCLC-Y was rich in genetic signatures associated with cytotoxic T cells, NK cells, and interferon signaling, suggesting a potential benefit for ICIs (59, 118).

Nevertheless, molecular subtypes have limitations in predicting the clinical effectiveness of ICI monotherapy for SCLC. In CheckMate 032, Rudin et al. reported that no statistically significant differences in PFS and OS were observed among 286 patients with advanced or metastatic SCLC divided into SCLC-A, SCLC-N, SCLC-P, and SCLC-Y subtypes based on baseline tumor tissues, whether in the Nivolumab or Nivolumab + Ipilimumab treatment group (48). Conversely, in the ICI-combo, SCLC-Y or -I subtype displayed efficacy-predicting potential. In IMpower133, Gay et al. (51) found that patients with SCLC-I subtype achieved the longest mOS after Atezolizumab + EP treatment (18.2 vs. 10.9 vs. 10.6 vs. 9.6 months) and obtain the best survival benefit from Atezolizumab + EP treatment compared with placebo + EP treatment (7.8 vs. 0.3 vs. 1.5 vs. 3.6 months). However, no significant difference in survival time among the four subtypes was observed in the placebo + EP arm, accentuating SCLC-I as an efficacy-predictive biomarker for ICI-combo, rather than a prognostic marker. Interestingly, patients with SCLC-P subtype had the shortest mOS in both Atezolizumab + EP arm and placebo + EP arm, indicating an association between SCLC-P subtype and poor prognosis. IMpower133 reported recently that transcriptomic analyses and non-negative matrix factorization of 271 pre-treatment patient tumor samples identified four subsets with general concordance to previously reported SCLC subtypes (SCLC-A, -N, -P, and -I). In particular, the authors uncovered two subsets with NE versus non-NE phenotypes and different clinical outcomes. Atezolizumab combined with Etoposide plus Carboplatin (EC)–treated tumors showed similar OS compared to placebo plus EC [HR, 0.85 (95% CI, 0.53–1.37)] in the effector T-cell (T-eff)–high/tumor-associated macrophage (TAM)–high non-NE tumors but markedly longer OS than placebo plus EC [HR, 0.26 (95% CI, 0.12–0.57)] in the T-eff–high/TAM-low NE tumors (52). Consistently, CASPIAN identified that both SCLC-Y and SCLC-I subtypes achieved the longest mOS benefit from Durvalumab + EP treatment than A, N, and P subtypes (10.4 vs. 0.8 vs. 2.4 months vs. no benefit, 6.3 vs. 1.2 vs. 4.1 months vs. no benefit, respectively) (119). Furthermore, patients with SCLC-P subtype consistently had the lowest mOS in the Durvalumab + EP treatment arm and the placebo + EP treatment arm. These results further validated the potential effectiveness of SCLC-I or SCLC-Y in predicting the efficacy of ICI-combo and SCLC-P in predicting poor prognosis in SCLC.

To overcome the difficulty of tumor tissue acquisition, long experimental period and high costs, studies explored IHC for SCLC subtyping and yielded inconsistent results. Baine et al. (53) analyzed 174 SCLC samples using IHC and found that ASCL1 was dominantly expressed in 69% samples, NEUROD1 in 17%, and POU2F3 in 7%, whereas no sample exhibited positive expression of YAP1 (H-score > 10), which was conflict to RNA-seq analysis. In a retrospective study in 28 patients with relapsed SCLC receiving ICI treatment, high POU2F3 expression (H-score > 90) was related to an increased ORR (AUC = 0.813) and prolonged OS (P = 0.022) (54), differing from findings of IMpower133 and CASPIAN, in which POU2F3 was a potential biomarker of poor prognosis after ICI-combo. This inconsistency may be attributed to the difference of therapeutic regimen. Additionally, inconsistency might lie in predicting efficacy of ICI-combo. Shirasawa et al. (120) retrospectively identified no significant difference in mPFS among pSCLC-A (n = 20), pSCLC-N (n = 8), and pSCLC-P (n = 5) using IHC in 34 patients with ES-SCLC with Atezolizumab + chemotherapy treatment. Furthermore, four of the six cases identified as SCLC-I based on RNA-seq were classified as pSCLC-A by IHC, and the other two cases as pSCLC-N and pSCLC-P, respectively, underscoring the discrepancies between IHC subtyping and RNA-seq subtyping.

Whether transcriptional subtype is a potential biomarker for immunotherapy in SCLC remains elusive. Firstly, methodology needs to be settled down because IHC data are conflicting with those via RNA-seq. Secondly, high heterogeneity of SCLC limits the predictive role of subtypes such as multiple subtypes coexisting within the same case (56, 121). Notably, previous results steamed from *post-hoc* analyses and further prospective clinical trials are urgently needed.

##### 2.2.2.2 Multi-omics integration

A major obstacle in SCLC is the lack of tumor samples that can be used for detailed molecular characterization, especially the multi-omics analysis of genomic, transcriptome, and proteome integration. Also, SCLC tumors are highly heterogeneous, and multi-omics at spatial resolution or single-cell analysis of the whole tumor tissues overcome the heterogeneity of tumors and tumor microenvironment. Recently, Liu et al. (55) performed an integrated genomic, transcriptomic, proteomic, and phosphoproteomic analysis of 112 treatment-naïve primary SCLC tumors and paired normal adjacent tissues from surgical resection. Unsupervised clustering divided SCLC tumors into four subtypes with biological differences and various therapeutic vulnerabilities. Protein genomics analysis enabled the immune landscape of SCLC to be characterized and revealed three immune clusters, including hot-tumor–enriched, cold-tumor–enriched, and normal adjacent tissue (NAT)–enriched subtypes. A total of 84.8% (84/99) of tumors belonged to immune-cold subtype tumor and were associated with worse prognosis (P = 0.0057). The frequency of ZFHX3 mutation was 19%, which was enriched in immune hot tumors and associated with immunogenicity. Patients with ZFHX3 mutation appeared to have better survival than patients with ZFHX3-WT (P = 0.073). ZFHX3 mutation was linked with immune activation behavior and the benefit for immunotherapy. Of the 12 patients with SCLC from two ongoing phase II trials (NCT04539977 and NCT04542369) who received neoadjuvant PD-1/PD-L1 blockade combined with chemotherapy, five had major pathologic response (MPR) and seven had non-MPR. The results of WES on the pre-treatment tissues showed that all three patients with ZFHX3 mutation (100%) belonged to MPR, whereas only two patients (22%) had MPR in WT patients (P = 0.045). The number of residual tumor cells in the ZFHX3 mutation group was significantly lower than that in the ZFHX3-WT group (P < 0.05). ZFHX3 mutation was associated with higher immune response and might be a potential predictive biomarker in patients with SCLC receiving immunotherapy. Similarly, the exploratory analysis of NSCLC treated with immunotherapy cohorts also demonstrated that ZFHX3 mutations are an independent predictive biomarker (122).

At present, the multi-omics study of SCLC is relatively limited, and only the integrated analysis of large samples by surgical resection has been reported. More exploration of advanced tumors is needed for SCLC, most of which are extensive stage at the time of diagnosis (123). Likewise, the lack of clinical treatment data limits the study of the correlation between multi-omics subtyping and treatment sensitivity.

##### 2.2.2.3 Inflammatory features of tumor-associated immune cells

T-cell inflamed GEP consisted of 18 inflammation-related genes, which involved in key downstream signaling molecules activated by IFN-γ (signal transducer and activator of transcription 1 (STAT1) and chemokine-like receptor 1 (CMKLR1)), chemokines (CXCR6, CCL5, and CXCL9), costimulatory receptors (CD27), HLA molecules induced by IFN-γ (HLA.DQA1 and HLA.DRB1), antigen-presenting machinery (PSMB10), checkpoint inhibitors upregulated by T-cell activation and IFN-γ signaling (PD-L1, PD-L2, IDO1, TIGIT, CD276, and LAG3), NK cell biology (HLA-E and NKG7), and CD8+ T cells (CD8A), providing a more comprehensive gene-level characterization of TME (124) and predicting the efficacy of immunotherapy in 22 cancer types (125). KEYNOTE-028 confirmed higher GEP score in patients including 24 patients with SCLC achieving prolonged PFS; regression meta-analysis on 14 cohorts showed that the GEP score was significantly associated with ORR (P = 0.012, n = 203) and PFS (P = 0.017, n = 203) (40). KEYNOTE-604 showed that patients with high GEP expression (>1st tertile values) receiving Pembrolizumab combined chemotherapy had longer mDOR than those receiving chemotherapy alone (5.55 vs. 4.11 months). Nevertheless, the mOS and mPFS of patients with high GEP expression were significantly longer than those with low GEP expression in both combination (P = 0.003 and P = 0.002, respectively) and chemotherapy group (P = 0.0002 and P = 0.001, respectively) (33). However, GEP as an efficacy-predicting marker for ICI alone or ICI-combo remains further verification in patients with SCLC.

##### 2.2.2.4 Single-cell transcriptional profiling

Single-cell omics technology can draw a comprehensive picture of tumor cells, immune cells, and stromal cells; elucidate the dynamic plasticity of a single cell; reveal the action network of cells with different phenotypes coexisting; and finally achieve the breakdown of tumor cells (126). It was found that chemotherapy promoted the remodeling of the extracellular matrix by fibroblasts and regulated the anti-tumor immune response of interferon-mediated B cells and T cells in the tissues of five patients with SCLC with chemotherapy (127), suggesting that the characteristics of the immune microenvironment may be used as a biomarker for predicting treatment efficacy. Analysis of scRNA-seq data of seven patients with treatment-naïve SCLC and 10 patients with SCLC treated with chemotherapy and ICI (Ipilimumab or Atezolizumab) revealed that the treated SCLC cells contained 14.2% non-NE cells, whereas the treatment-naïve cells contained 1.1% non-NE cells. Compared with NE cells expressing ASCL1 and TFF3, heterogeneous non-NE cells showed the overexpression of IFITM3, B2M, ANXA4, VIM, CD74, S100A11, and YAP1 and the activation of interferon signaling, cell cycle, and antigen processing (128), providing clues that scRNA-seq indicated the benefit of immunotherapy.

The high-precision single-cell transcriptomic analysis of ~5,000 individual cells from primary tumors (PTs) and matched NATs from 11 patients with SCLC revealed that the differentially expressed genes (DEGs) of immune expression patterns not only showed more pronounced intra-tumor heterogeneity (ITH) but also were associated with different immune checkpoint blockade responses. These DEGs containing 53 known genes that reflect immune signature, including HLA-related genes, antigen-presentation–related genes, DNA repair genes, and chemokines, which can represent the immune status of tumors. Among which, HLA genes, including HLA-A, HLA-B, HLA-C, HLA-DMA, and HLA-DRB1, have vital functions in the immune system and immune response, such as antigen presentation (129), immune surveillance (130), and immune regulation (129). The targeted gene expression analysis of the above DEGs in 14 tumors of patients with SCLC who received anti–PD-1 treatment improved that four patients with “immune hot” features tended to benefit more from ICI than the other patients (n = 10) with “immune cold” SCLC (56). A few existing articles on single-cell sequencing of patients with SCLC suggest that immune-related differences from single-cell resolution may be used to help select patients who might benefit from these promising strategies, which is worthy of validation in large cohorts.

#### 2.2.3 Tumor ITH

ITH is defined as an uneven distribution, spatially or temporally, of genomic diversification in an individual tumor, fostered by accumulated genetic mutations (131), which was associated with the poor prognosis in solid tumors (132, 133). It has recently been reported that ITH, manifested by the distribution of clonal versus sub-clonal mutations and neoantigens (134), may influence immune surveillance (135–137).

In SCLC, Wang et al. (56) performed high-precision scRNA-seq and low-pass WGS of PTs and matched normal adjacent tissues from 11 patients with SCLC and revealed that patients with ITH had shorter disease-free survival (log-rank P = 0.0489) and a higher risk of recurrence (log-rank P = 0.0371). The IHC of tissue microarray also demonstrated that heterogeneous subtypes were associated with poor OS (log-rank P = 0.0455), suggesting that ITH may also be an immunotherapy biomarker in SCLC worthy of investigation, but its association with immunotherapy needs to be determined first.

## 3 Liquid biomarkers

Tissue limitation has been one of the main reasons limiting translational studies in SCLC. SCLC is often associated with high level of circulating tumor DNA (ctDNA) and CTC. Therefore, liquid biopsy strategies have been extensively researched in this setting (**Table 6**).

**Table 6:** Studies of possible liquid biomarkers for immunotherapy in SCLC.

Biomarker	N	Setting	Cutoff	Treatment	DCR	Median PFS	Median OS	Reference
ACS	126	First-line	1.115	Immunotherapy combined with chemo or chemo alone	NA	ACS high vs. low: 7.7 vs. 4.9 months, P = 0.007	ACS high vs. low: 16.3 vs. 13.6 months, P = 0.033	(138)
LIPI	100	First-line	dNLR: 4.0 U/L; LDH: 283 U/L LIPI good: dNLR < 4.0 and LDH < 283 U/L LIPI intermediate: dNLR < 4.0 and LDH ≥ 283 U/L, or dNLR ≥ 4.0 and LDH < 283 U/L; LIPI poor: dNLR ≥ 4.0 and LDH ≥ 283 U/L	PD-1/PD-L1 inhibitors plus chemo	NA	LIPI good vs. intermediate/poor: 8.4 vs. 4.7 months, p = 0.02	LIPI good vs. intermediate/poor: 23.8 vs. 13.3 months, p = 0.0006	(139)
LMR	236	First-line	3.26	ICI + EP; EP	NA	NA	Independent prognostic factors for OS (HR, 0.54; P = 0.049)	(140)
NLR			3.1			Independent prognostic factors for PFS (HR, 0.45; P = 0.028)	NA	
PLR	53	First-line	119.23	Chemotherapy and Atezolizumab.	NA	Low vs. high: 6 months of PFS: 50% vs. 22%, P = 0.014,	Low vs. high: 1-year OS: 87% vs. 42%, P = 0.0004 independent prognostic factors for OS (HR 4.63, P = 0.05	(141)
ctDNA (5 genes)	68	First-line	At least one somatic mutation	Atezolizumab or chemo	Detectable vs. undetectable in immunotherapy group: 13.3% vs. 50%, P = 0.0145	NA	NA	(142)
ctDNA (58 genes)	33	First-Third lines	Mutant allele fraction of tumor-derived mutations and plasma aneuploidy	Chemotherapy ± Atezolizumab or Durvalumab + Ipilimumab	NA	Elimination of total cfTL vs. recrudescence or persistent cfTL: not reached vs. 6.18 vs. 1.74 months, P < 0.00001	Elimination of total cfTL vs. recrudescence or persistent cfTL: not reached vs. 12.35 vs. 6.48 months, P = 0.0006	(143)
ctDNA (hTERT)	46	First-line	Log cfDNA levels 7.650 for PFS and 8.077 for OS analyses	Chemo ± Immunotherapy	NA	HR, 5.06; 95% CI (1.89–13.6), P = 0.001	HR, 3.32; 95% CI (1.50–7.37), P = 0.003	(144)
IL-2	84	First-line	≥ median value	Chemo alone or combined with Ipilimumab	NA	NA	ICI-combo: 30.5 vs. 8 months, P = 0.015 Chemo: 12.2 vs. 12.6 months, P = 0.273	(145)
IL-6			< median value				ICI-combo: 18.5 vs. 9.5 months, P = 0.026 Chemo: P = 0.073	
TNF-α							ICI-combo: 18.5 vs. 7.8 months, P = 0.004 Chemo: P = 0.222	

DCR, disease control rate; PFS, progression-free survival; OS, overall survival; ACS, aneuploid CTC score; LIPI, lung immune prognostic index; dNLR, derived neutrophil-to-lymphocyte ratio; LMR, lymphocyte-to-monocyte ratio; NLR, neutrophil‐to-lymphocyte ratio; PD-1, programmed cell death protein 1; PD-L1, programmed cell death-ligand 1; ICI, immune checkpoint inhibitor; EP, Etoposide plus Cisplatin; HR, hazard ratio; PLR, platelet-to-lymphocyte ratio; ctDNA, circulating tumor DNA; cfDNA, circulating free DNA; cfTL, cell-free tumor load; ICI-combo, ICI + chemotherapy; chemo, chemotherapy. NA, not applicable.

### 3.1 CTCs

Another study indicated that, in a cohort of 82 patients with breast cancer receiving ICI plus chemotherapy, continuous sampling of CTCs was conducted, and it was found that the patients with ≥2 CTCs per 7.5 mL at baseline were positively correlated with the tumor inflammation signature, and the CTC status was most significantly correlated with the treatment outcome 4 weeks after treatment, suggesting a potential of using CTCs as an accessible biomarker source in patients with breast cancer treated with immunotherapy (146). Patients with SCLC with a higher baseline aneuploid CTC score (ACS) (ACS > 1.115) had longer mPFS (7.7 months, P = 0.007) and mOS (16.3 months, P = 0.033) in immunotherapy combined with chemotherapy than those with chemotherapy alone (mPFS, 4.9 months; mOS, 13.6 months), demonstrating the utility of CTC detection in SCLC risk stratification and treatment response monitoring (138). In the future, larger-scale prospective randomized or controlled trials need to be designed and elucidate the role of liquid samples from patients with SCLC in the benefit of immunotherapy efficacy.

### 3.2 Ratios of cells in the blood

The lung immune prognostic index (LIPI) consists of derived neutrophil-to-lymphocyte ratio (dNLR) and lactate dehydrogenase (LDH) (147). Li et al. (139) divided LIPI in patients with SCLC into two groups (good and moderate/poor), those with dNLR < 4.0 and LDH < 283 U/L were evaluated as good LIPI group, those with dNLR < 4.0 and LDH ≥ 283 U/L or dNLR ≥ 4.0 and LDH < 283 U/L were evaluated as moderate LIPI group, and those with dNLR ≥ 4.0 and LDH ≥ 283 U/L were evaluated as poor LIPI group. The results showed that the mPFS and mOS of patients in the good LIPI group were better than those in the medium/poor LIPI group (mPFS: 8.4 vs. 4.7 months, P = 0.02; mOS: 23.8 vs. 13.3 months, P = 0.0006). Multivariate Cox regression analysis showed that pretreatment LIPI was an independent prognostic indicator for OS in patients with ES-SCLC treated with first-line PD-1/PD-L1 inhibitors combined with chemotherapy.

Multivariate Cox regression analysis (140) revealed that neutrophil-to-lymphocyte ratio (NLR) was an independent predictor of PFS in patients [HR = 0.45, 95% CI (0.22, 0.92), P = 0.028]; lymphocyte-to-monocyte ratio (LMR) was an independent predictor of OS in patients [HR = 0.54, 95% CI (0.30, 0.99), P = 0.049]; whereas LIPI, systemic immune-inflammatory index (SII), platelet-to-lymphocyte ratio (PLR), systemic inflammatory response index (SIRI), and prognostic nutritional index (PNI) were not independent predictors of PFS and OS in patients with ES-SCLC. QI et al. (141) found that patients with baseline PLR > 119.23 had significantly shorter OS than patients with PLR ≤ 119.23 in patients with ES-SCLC receiving first-line atezolizumab combined with chemotherapy, and multivariate analysis indicated that PLR was the only independent prognostic factor for OS [HR = 4.63, 95% CI (1.00, 21.46), P = 0.05], whereas LMR, NLR, PNI, SII, and SIRI were not independent predictors of PFS and OS in patients with ES-SCLC (**Table 2**).

### 3.3 ctDNA

The limited availability of tumor tissue and strong heterogeneity in SCLC present challenges for the clinical application of tissue-derived biomarkers (148). Liquid biopsies, particularly those utilizing ctDNA harboring cancer-specific genetic information and are considered as a surrogate for tumor DNA (149), offer a non-invasive, spatially homogeneous, and real-time approach in various cancer types (150, 151).

Herbreteau et al. observed that patients with ctDNA levels exceeding the median variant allele fraction (VAF) had poorer OS (5.3 vs. 10 months) and PFS (2.1 vs. 12.5 months), suggesting the prognostic potential of ctDNA (142). A prospective study by Smith et al. collected 75 serial plasma samples from 25 patients with SCLC and demonstrated a significant positive correlation between mean VAF and total body tumor volume, particularly in treatment-naive and pretreatment samples (152). For the immunotherapy, the authors retrospectively examined five genes in baseline ctDNA by NGS from 46 patients with SCLC receiving Atezolizumab or chemotherapy (142) and found that patients with detectable ctDNA (at least one somatic mutation) exhibited a significantly lower disease control rate compared to those without detectable ctDNA in immunotherapy group but not in chemotherapy group (13.3% vs. 50%, P = 0.0145). A similar data were identified in a recent retrospective study on 171 serial plasma samples from 33 patients with metastatic SCLC treated with chemotherapy or immunotherapy regimens by targeted error-correction sequencing (Tec-seq) of 58 genes (143). Patients who achieved sustained complete elimination of total cell-free tumor load (cfTL), assessed through a combination of mutant allele fraction of tumor-derived mutations and plasma aneuploidy, had longer mOS and mPFS compared with patients who had recrudescence or persistent cfTL (OS not reached vs. 12.35 vs. 6.48 months, P = 0.0006; PFS not reached vs. 6.18 vs. 1.74 months, P < 0.00001). In addition, Macía et al. evaluated ctDNA levels by detecting the telomerase reverse transcriptase single-copy gene of 111 plasma samples from 46 patients with SCLC treated with chemotherapy or ICI-combo. Patients with baseline and 3-week log ctDNA levels below cutoff value achieved longer OS and PFS (144). It is worth noting that, whereas Herbreteau et al. found the ability of ctDNA to predict immunotherapy efficacy but not chemotherapy, other studies reported ctDNA’s predictive value for therapeutic response independently of the treatment regimen (153, 154).

Although these studies provide new perspectives for ctDNA as a biomarker of clinical efficacy in SCLC (**Table 3**), ctDNA monitoring still faces challenges such as the lack of a definitive gene panel, the criteria to determine ctDNA levels, and the superior method, including NGS and droplet digital PCR (153, 155, 156). In conclusion, further research is necessary to address these salient issues in order to demonstrate the immense promise of ctDNA as a predictive biomarker of immunotherapy efficacy in SCLC.

### 3.4 Exosomal miRNAs

Exosomes are endocytosis-derived vesicles with 30–150 nm in size that play important role in cell-to-cell communication, as well as transporting various cell-derived molecules including proteins, lipids, DNA, mRNA, and microRNAs (miRNAs) (157, 158). Previous data explored that exosomes were involved in various stages of tumor progression, including immunomodulation, angiogenesis, metastasis, drug resistance, and cell proliferation (159, 160). The significantly differential exosomal miRNAs in patients with lung cancer were identified in a study using serum samples including 11 patients with NSCLC, nine patients with SCLC, and 10 healthy controls. The content of exosomal miRNA not only can accurately distinguish patients with SCLC and NSCLC but also aid in monitoring the progress treatment (161). Three-miRNA panel (miR-200b-3p, miR-3124-5p, and miR-92b-5p) in serum was significantly associated with a poorer prognosis (P = 0.0029) and may serve as a diagnostic and prognostic marker for SCLC (162). Notably, both exosomal miR-1290 and miR-29c-3p displayed substantial discriminatory capacity in distinguishing between NSCLC and SCLC, as indicated by their respective area under curve (AUC) values of 0.810 and 0.842 (163). Further studies reported that exosomal miR-1228-5p and miR-375-3p in SCLC can promote the proliferation and migration of SCLC cells by targeting DUSP22 or claudin-1, respectively (164, 165). Exosomal miR-15a-5p/CCNE1 axis and miR-92b-3p also involved in chemoresistance of SCLC (166, 167). Moreover, exosomal miRNAs may be related to the therapeutic efficacy of SCLC immunotherapy or immune combination therapy with regard to the occurrence, development and chemoresistance, immunotherapy resistance (168), and the formation of immunosuppressive microenvironment (169, 170). Overall studies demonstrated that 43% of the RNA in exosomes contain miRNA, emphasizing the importance of miRNA in exosome function (171). Exosomal miRNAs in blood have the potential to be a breakthrough biomarker for SCLC immunotherapy, but more studies are needed to correlate blood exosomal miRNAs with the efficacy of SCLC immunotherapy, and identifying generalized panel-based miRNA detection methodology is a critical point in achieving practical application.

### 3.5 Proteins in blood

Plasma proteins and metabolites also have potential predictive value. In two independent prospective tumor cohorts treated with ICIs (discovery cohort, n = 95; validation cohort, n = 292), it was found through the Olink^®^ plasma proteome that the level of leukemia inhibitory factor (LIF) in baseline blood had a good predictive value for therapeutic benefit (training set, AUC = 0.735; validation set, AUC = 0.622) (172). Karlsson et al. analyzed the plasma proteins of 109 patients with melanoma receiving targeted therapy or immunotherapy before and during treatment through plasma untargeted proteomics platform and constructed a panel of inflammation and apolipoproteins as prognostic and predictive biomarker, to improve the treatment options for patients with cutaneous melanoma (173). The plasma proteins of patients with SCLC are still in the initial exploration stage (174, 175), and the above studies provide ideas for the research direction of SCLC.

Increasing studies showed the role of cytokines in response to varied treatment in SCLC (51, 176, 177). Welbin et al. (145) evaluated the level of several cytokines in the baseline serum of patients with SCLC receiving chemotherapy alone (n = 47) or combined with Ipilimumab (n = 37) and found that mOS of patients with high baseline IL-2 expression (≥ 1.65 pg/mL) (n = 15) was significantly longer than patients with low-expression (n = 20) (30.5 vs. 8 months, P = 0.015) in the ICI-combo cohort but not the chemotherapy cohort (12.2 vs. 12.6 months, P = 0.273), suggesting that the baseline IL-2 level in patients with SCLC can predict the benefit of ICI-combo. Interestingly, patients with low expression levels of IL-6 or TNF-α had longer mOS than those with high expression levels (18.5 vs. 9.5 months, P = 0.026; 18.5 vs. 7.8 months, P = 0.004, respectively) in the ICI-combo cohort but not in the chemotherapy cohort (P = 0.073 and P = 0.222, respectively), suggesting the predictive role of low IL-6 and TNF-α ICI-combo benefits in SCLC.

Serum PD-L1 (sPD-L1) is involved in immunosuppression and resistance to ICI therapy and shows higher levels in the serum of cancer patients (178). Although sPD-L1 was considered as a potential marker for poor prognosis in patients with SCLC treated with chemotherapy (179), the analysis of sPD-L1 levels in patients with various solid tumors treated with ICI (Ipilimumab) revealed no relevant differences in sPD-L1 levels before and after immunotherapy in patients with SCLC, excluding sPD-L1 as a predictive biomarker of response to therapy with Ipilimumab (178). Exosomes in blood samples are also indispensable exploration directions as biomarkers related to immunotherapy in tumors. The expression of PD-L1 in blood exosomes has been reported to have predictive value for immunotherapy. In patients with metastatic melanoma, the level of circulating exosome PD-L1 could serve as an indicator of the adaptive response of tumor cells to T-cell activation, stratifying clinical responders and non-responders (180). The combination of radiomics and PD-L1 detection in plasma extracellular vesicle (EV) had high sensitivity and specificity in identifying patients with NSCLC with non-response to ICIs and outperformed tissue PD-L1 (181), which could more effectively predict the efficacy and prognosis of patients with immunotherapy. Current studies have shown the role of EVs in the diagnosis and prognosis of SCLC (182, 183), chemotherapy resistance (184), and the cross-talk mechanism between EV and immune microenvironment in SCLC (185, 186), but the research on EV as a biomarker related to the immunotherapy response in SCLC is relatively blank.

## 4 Discussion

Immunotherapy, as another revolution after targeted therapy, has opened a new chapter in the treatment of ES-SCLC. IMpower133 and CASPIAN firstly demonstrated the efficacy of PD-L1 inhibitors combined with chemotherapy, followed by CAPSTONE-1 and PD-1 inhibitors in SCLC demonstrated by ASTRUM-005, RATIONALE-312, and EXTENTORCH, to the innovative four-drug model of ETER701, which further refreshed the OS survival of first-line treatment of ES-SCLC to 19.3 months (17).

Novel targeted drugs and new combination strategies are important direction of SCLC translational and clinical research. Lurbinectedin breakthrough past 20 years obstacle of only chemotherapy in second-line SCLC and enhance the anti-tumor immune response by regulating the tumor microenvironment. Therefore, the hypothesis of combination of immunotherapy and Lurbinectedin after first-line therapy as a strategy to improve the efficacy of SCLC was tested in numbers of clinical studies, including LUPER and 2SMAL. Immune cell–based strategies are also ongoing, including a phase II study of Monalizumab in combination with Durvalumab and chemotherapy for the first-line treatment of ES-SCLC derived from the mechanism that Monalizumab restores NK cell and T-cell function. In addition to strategies to improve NK cell and T-cell function, adoptive NK cells or CAR-NK cells, as well as CAR-T therapies, are also being carried out in SCLC.

ADC drugs are another promising research direction and hot spot in SCLC including B7-H3 and TROP2. The Ideate-lung01 is a phase II clinical trial using I-DXd for patients with recurrent SCLC who were divided into 8 mg/Kg (n = 46) arm and 12 mg/Kg (n = 42) arm, respectively. The ORR was 26.1% and 54.8%, PFS was 4.2 and 5.5 months, OS was 9.4 and 11.8 months, and then 12 mg/Kg was selected as the phase 3 study dose (187). Currently, a phase III study of DS-7300 in recurrent SCLC also been initiated. In addition, ADCs targeting SEZ6 also showed excellent potential, such as ABBV-706, which included 23 patients with SCLC in the first-in-human study, with an ORR of 60.9%. In addition, the rise of bispecific antibody drugs will bring hope for the treatment of SCLC. PM8002 is a bispecific antibody drug targeting PD-L1 and VEGF-A, and, in a phase II study, PM8002 combined with paclitaxel showed 81.8% DCR, 72.7% ORR, and up to 5.5 months mPFS in the second-line SCLC (n = 27) who failed in first-line platinum-based chemotherapy with or without ICIs, implying encouraging anti-tumor activity. The clinical trials targeting Trop2 ADC in SCLC are currently underway; phase I and II data showed that, for recurrent SCLC, ORR is 30%–40%, PFS is around 4 months, and the preliminary OS is 13.6 months with manageable safety profile, so that Trop2 ADC is worthy further research in SCLC (188, 189).

It is also one of the future directions to study how to accurately formulate a combination drug regimen according to the genetic characteristics of individual patients, tumor stage, and other factors. Through multi-omics analysis such as genomics and proteomics of a large number of patient samples, potential biomarkers are discovered, which can help achieve early diagnosis, accurate staging, and personalized treatment. There have been some studies on PD-L1 expression as biomarkers for SCLC immunotherapy, and there are many more undiscovered biomarkers waiting to be explored. Recently, progression in defining transcriptome molecular subtypes and characterizing immune microenvironment provides multiple new potential biomarkers for SCLC. Subtype classifications based on transcription factor expression and immune infiltration may be a prerequisite for the development of biomarkers, as SCLC subtypes with high immune infiltration and high immune response-related gene expression are more likely to benefit from ICI therapy in the exploratory studies of clinical trials. Furthermore, accurate biomarker research on the mechanism of different regiments is needed, which will improve the application and efficacy of targeted therapy drugs, including immunotherapy, in SCLC.

In the future, individualized therapeutic regime should be developed on the basis of the genotyping of patients with SCLC. In addition, patients with different genetic variants may respond differently to the treatment, for example, patients with certain genetic variants may be more sensitive to the targeted drugs. It is particularly important to conduct translational research on precision diagnosis and treatment in multiple dimensions. With the continuous development of genetic testing technology, there will be more research and application opportunities for this genotyping-based precision therapy.
